# Strengths and pitfalls of NNT and NNH in early breast cancer escalation trials

**DOI:** 10.1016/j.breast.2026.104820

**Published:** 2026-05-29

**Authors:** Roberto Buonaiuto, Federica Pia Mangiacotti, Fabiola Giudici, Margherita Tafuro, Roberta Scarfetta, Alessandra Longobardi, Martina Pagliuca, Aldo Caltavituro, Luca Arecco, Evandro de Azambuja, Mario Giuliano, Grazia Arpino, Lucia Del Mastro, Carmine De Angelis, Michelino De Laurentiis, Fabio Puglisi

**Affiliations:** a Clinical and Translational Oncology, Scuola Superiore Meridionale, Via Mezzocannone 4, Naples, 80134, Italy; b Department of Breast and Thoracic Oncology, Istituto Nazionale Tumori IRCCS “Fondazione G. Pascale”, Via Mariano Semmola 52, Napoli, 80131, Italy; c Université Libre de Bruxelles (ULB), Hôpital Universitaire de Bruxelles (H.U.B), Institut Jules Bordet, Bruxelles, Belgium; d Oncology Unit, Department of Clinical Medicine and Surgery, University of Naples Federico II, Via Sergio Pansini 5, Naples, 80131, Italy; e Unit of Cancer Epidemiology, CRO Aviano, National Cancer Institute, IRCCS, Via Franco Gallini 2, Aviano, 33081, Italy; f Medical Oncology, Fondazione Policlinico Universitario Campus Bio-Medico, Via Alvaro Del Portillo 200, Roma, 00128, Italy; g Molecular Predictors and New Targets in Oncology, Inserm U981, Université Paris-Saclay, Gustave Roussy, 114 Rue Edouard Vaillant, Villejuif, 94800, France; h Department of Medical Oncology, IRCCS Ospedale Policlinico San Martino, Largo R. Benzi 10, Genova, 16132, Italy; i Dipartimento di Medicina Interna e Specialità Mediche (DIMI), University of Genova, Viale Benedetto XV 6, Genova, 16132, Italy; j Department of Medical Oncology, CRO Aviano, National Cancer Institute, IRCCS, Via Franco Gallini 2, Aviano, 33081, Italy; k Department of Medicine, University of Udine, Via Palladio 8, Udine, 33100, Italy

**Keywords:** Number needed to treat, Number needed to harm, Early-stage breast cancer

## Abstract

**Background:**

The management of patients with high-risk early breast cancer (eBC) requires balancing between clinical benefit and toxicity. Number Needed to Treat (NNT) and Number Needed to Harm (NNH) may provide a clinically intuitive framework to assess benefit–risk profiles, but their use in eBC has not been systematically explored.

**Methods:**

We analysed 10 phase II–III randomized clinical trials (RCTs) of treatment escalation strategies in patients with mid to high-risk eBC. To ensure consistency and cross-trial comparison, invasive disease-free survival (IDFS), disease-free survival (DFS), event-free survival (EFS), and overall survival (OS) were extracted at 3 and 5 years (y). Safety outcomes included any-grade (G) and G ≥ 3 adverse events (AEs), G5 AEs, and treatment discontinuation (TD). Absolute risk reduction (ARR) and absolute risk increase (ARI) were used to calculate NNT and NNH, respectively, reported with 95% CIs.

**Results:**

In patients with the HER2+ eBC, KATHERINE showed the most favorable efficacy, with NNTs of 9 and 8 for IDFS at 3 and 5 years, respectively, and a NNT of 27 for OS at 5 years. APHINITY reported the most favorable safety profile, with the highest NNHs for G ≥ 3 AEs (15) and TD (80). In patients with TNBC, CREATE-X reported the lowest NNTs for DFS (7 at both 3 and 5 years). The most favorable results in OS were observed in OlympiA (NNT 26) and KEYNOTE-522 (NNT 20). Regarding safety, KEYNOTE-522 achieved the most favorable NNH for G ≥ 3 AEs (26), while OlympiA showed the best NNH for TD (16). In patients with HR+/HER2− eBC, OlympiA showed the strongest efficacy results, with the lowest NNTs for IDFS at 3 (10) and 5 years (9), as well as for OS at 5 years (26). The most favorable NNHs were reported for G ≥ 3 AEs in OlympiA (8) and for TD in monarchE (19).

**Conclusion:**

NNT and NNH provide intuitive metrics to communicate benefit–risk trade-offs in high-risk eBC, although their variability across trials, subgroups, and timepoints requires cautious interpretation.

## Highlights

• NNT and NNH quantify benefit–risk balance between treatment efficacy and toxicity.
• NNT and NNH may vary substantially by trial design, endpoints and follow-up duration.
• These simple metrics may support clearer communication and shared decision-making.

## 1 Introduction

The management of early breast cancer (eBC) requires a careful balance between maximizing the therapeutic efficacy and minimizing treatment-related toxicity in the adjuvant setting. Patients identified as being at higher risk of recurrence, based on clinicopathological features such as nodal status, histological grade, Ki-67 index, and tumor size, as well as molecular profiling through multigene assays, are often candidates for treatment escalation strategies. These approaches aim to reduce the risk of recurrence by intensifying conventional therapies [1,2] or by integrating novel targeted agents [[1], [2], [3]]. However, the potential benefits of these strategies must be carefully balanced against the higher risk of adverse events (AEs), underscoring the complexity of clinical decision-making in this setting. Clearly communicating the expected benefits and potential toxicities of treatment interventions to patients is crucial in clinical practice, particularly for those with early-stage disease. In this context, simple and intuitive measures that express the absolute magnitude of both benefits and harms may be especially valuable in guiding shared decision-making.

The Number Needed to Treat (NNT) and Number Needed to Harm (NNH) are quantitative metrics that offer a structured framework for evaluating the benefit-risk profile of therapeutic interventions [[4], [5], [6], [7]]. NNT represents the number of patients who need to be treated to prevent one additional adverse clinical event, whereas NNH indicates the number of patients who must be treated for one additional patient to experience an AE. A lower NNT reflects greater treatment efficacy, with the theoretical optimal value of 1 indicating that every treated patient benefits from the new strategy, while no benefit is observed in the control group, while higher NNT values indicate reduced therapeutic effectiveness; in contrast, a lower NNH indicates a higher frequency of AEs, whereas higher NNH values reflect a lower risk of harm, corresponding to a more favorable safety profile. Although the NNT and the NNH provide a pragmatic framework for assessing the balance between efficacy and safety, these metrics are not without limitations. Their interpretation is highly context-dependent, influenced by the clinical setting, patient population, and temporal dynamics of treatment effects, and may therefore fail to fully capture the complexity of clinical outcomes.

In this study, we aim to critically appraise the application of NNT and NNH in the context of patients with high-risk eBC through a descriptive analysis of RCTs investigating treatment escalation strategies. By examining these metrics, our analysis seeks to elucidate their potential to inform clinical decision-making, while simultaneously underscoring their methodological constraints and interpretative challenges.

## 2 Methods

### 2.1 Research strategy, inclusion and exclusion criteria

We performed a descriptive, trial-level analysis of randomized phase II–III clinical trials evaluating therapeutic escalation strategies in early breast cancer (eBC). Treatment-escalation strategies for patients with intermediate-to high-risk eBC were identified from current international guidelines, including ESMO and NCCN [8,9]. For each strategy, we selected the pivotal randomized trial that supported EMA and/or FDA approval in the early breast cancer setting.

Specifically, we included trials that.

I. Evaluated escalation of systemic therapy in the (neo)adjuvant setting;
II. Enrolled patients with intermediate-to high-risk eBC, defined as stage II–III BC according to the AJCC Cancer Staging Manual, 7-8th Edition;
III. Investigated therapeutic agents that received regulatory approval and are currently recommended by international guidelines [8,9].

TRAIN-2 [10] and CREATE-X [11] were included despite not being registrational trials, as they established carboplatin-based chemotherapy and adjuvant capecitabine as treatment options currently recommended by international guidelines for HER2-positive and TNBC, respectively.

### 2.2 Data extraction

Two reviewers (RB, FM) independently extracted data using a standardized form. For each trial, we collected patient characteristics, median follow-up, and endpoints information, including type (primary vs secondary) and assessment timing (interim, final, or descriptive). Efficacy endpoints included invasive disease-free survival (IDFS), disease-free survival (DFS), event-free survival (EFS), and overall survival (OS). Safety data on adverse events (AEs) were collected, including any-grade AEs, grade ≥3 AEs, grade 5 events, AEs leading to treatment discontinuation (TD), and the most frequently reported grade ≥3 AEs.

### 2.3 Calculation of efficacy and toxicity metrics

Efficacy and toxicity measures were calculated for each trial using the most recent data available at the time of analysis. The Absolute Risk Reduction (ARR) was defined as the difference between the event (disease recurrence or death) rate in the control arm (CER) and those in the experimental arm (EER) (ARR = CER − EER). The Absolute Risk Increase (ARI) was defined as the difference in G ≥ 3 adverse events rate between the experimental arm (ETR) and the control arm (CTR) (ARI = ETR − CTR).

The NNT and NNH were derived as the reciprocals of ARR and ARI, being NNT = 1/ARR and NNH = 1/ARI, respectively.

For example, if the control recurrence rate is 0.20 and the treatment recurrence rate is 0.15, the ARR is 0.05, yielding an NNT of 1/0.05 = 20. For instance, if the adverse event rate is 0.08 with treatment versus 0.04 with control, the ARI is 0.04, yielding an NNH of 1/0.04 = 25.

To ensure methodological consistency and facilitate cross-trial comparisons, survival outcomes were assessed at standardized landmarks of 3 and 5 years. When survival probabilities were not directly reported, estimates were derived by reconstructing individual patient data (IPD) from latest available published Kaplan-Meier curves using the IPDfromKM Shiny app [12]. Reconstruction accuracy was considered acceptable when the root mean squared error (RMSE) was ≤0.05, the mean absolute error ≤0.02, and the maximum absolute error was ≤0.05 [12].

### 2.4 Confidence intervals for the NNTs and NNHs

The conventional method for calculating 95% confidence intervals (CIs) for NNTs/NNHs relies on the Wald method, which often yields overly narrow intervals. To address this limitation, we applied the Newcombe-Wilson Hybrid Method, which provides more accurate and informative 95% CIs estimates [[13], [14], [15], [16], [17]]. While the point estimate of the NNT (or NNH) is simply the reciprocal of the ARR or ARI, calculating its confidence interval is more complex, particularly when the 95% CI for ARR includes zero. In such cases, the 95% CI for NNT necessarily includes infinity (1/0 = ∞), implying that an infinite number of patients would be required to observe a benefit, a concept that is counterintuitive.

To improve interpretability, Altman et al. [18] recommended distinguishing between NNTB (Number Needed to Treat for Benefit) and NNTH (Number Needed to Treat for Harm). For example, if the ARR is 0.10 (95% CI: −0.05 to 0.25), the result would be expressed as NNTB of 10 (95% CI: NNTH = 20 to ∞ to NNTB = 4), making explicit that the true effect could plausibly range from harm to benefit.

In this example, the NNTH of 20 corresponds to the negative lower bound of the ARR confidence interval (−0.05) and indicates that, at one extreme, one additional patient may be harmed for every 20 patients treated, suggesting a potentially weaker or even detrimental treatment effect. The infinity (∞) reflects the crossing zero within the ARR CI, implying the possibility of no true difference between the treatment and control. The NNTB of 4 corresponds to the positive upper bound of the ARR CI (0.25) and suggests that, at the other extreme, only four patients would need to be treated for one additional patient to benefit, indicating a potentially stronger treatment effect. When the 95% CI of the ARR included zero, the corresponding NNT confidence interval was reported according to the Altman convention [18], in the following format: (−20 to ∞ to 4), distinguishing between the number needed to treat for benefit and the number needed to treat for harm. In this setting, the interval necessarily crosses infinity and indicates that the data are compatible with benefit, no effect, or harm. Therefore, these estimates were considered descriptive measures of uncertainty and were not interpreted as evidence of treatment benefit.

## 3 Results

Ten RCTs (KATHERINE [19], APHINITY [20,21], NeoSphere [22], TRAIN-2 [10], ExteNET [23], monarchE [24], NATALEE [25], CREATE-X [11], OlympiA [26] and KEYNOTE-522 [27]) were included in the analysis. In APHINITY [20,21], only patients with node-positive disease were considered, whereas in ExteNET [23] the analysis was restricted to the HR+/HER2+ subgroup who had completed adjuvant trastuzumab-based therapy within one year, in accordance with current international guidelines [8,28] and regulatory approval [29,30]. The detailed study characteristics, including treatment arms, are summarized in Table 1.

**Table 1:** Key study characteristics in phase II-III trials included in the analysis.

Year	Trial	Phase	Experimental arm	Control arm	Number of subjects in the Experimental arm	Number of subjects in the Control arm	Primary Endpoint	Secondary Endpoint	Median follow-up, months	Type of analysis primary endpoint	Type of analysis secondary endpoint
2025	KATHERINE [19]	3	T-DM1	Trast	743	743	IDFS	OS, DFS, DRFI, safety	101	Final	Interim analysis II (OS)
2024/2025	APHINITY (N+) [20,21]	3	Pert + Trast + CT	Plac + Trast + CT	1503	1502	IDFS	OS, DFS, RFI, DRFI, safety, QoL, IDFS^a^	100 (IDFS) 136 (OS)	Descriptive analysis	Final analysis (OS)
2024	KEYNOTE-522 [27]	3	Pembro + CT	Plac + CT	784	390	EFS, pCR	OS, pCR in ypT0 ypN0 and ypT0/Tis, pCR, EFS, and OS in the PD-L1 CPS ≥1 population, safety	75.1	Interim	Interim
2024	OlympiA HR + [26]	3	Ola	Plac	168	157	IDFS	DDFS, OS	73.2	Interim III	Interim III (OS)
2024	OlympiA HR- [26]	3	Ola	Plac	751	758	IDFS	DDFS, OS	73.2	Interim III	Interim III (OS)
2017	CREATE-X [11]	3	Cape	Plac	443	444	DFS	OS	42	Final	Final (OS)
2021	ExteNET HR+ ≤1 y [23]	3	Neratinib	Plac	670	664	IDFS	DFS, DDFS, TDR, incidence of first occurrence of CNS, OS, safety	96 (OS)	Final	Final (OS)
2025	monarchE [31]	3	Abema + ET	ET	2808	2829	IDFS (STEEP criteria)	DRFS, OS, safety, PRO, PK	76.2	Descriptive	Interim (OS)
2025	NATALEE [25]	3	Ribo + ET	ET	2549	2552	IDFS (STEEP criteria)	RFS, DDFS, OS, safety, PK	55.4 (IDFS), 56.5 (OS)	Prespecified time triggered analysis	Descriptive analysis (OS)
2021	TRAIN-2 [10]	3	PTC + Pert	FEC + Trast + Pert	219	219	CR	EFS, OS, safety	36	Interim I	Interim II (EFS)
2016	NeoSphere [22]	2	Trast + Doce (A)	Trast + Pert + Doce (B) Trast + Pert(C) Pert + Doce(D)	101 (B)	103(A) 96(C) 92(D)	pCR	PFS, DFS	60	Final	Final

Abbreviations: A, arm A; Abema, abemaciclib; AE, adverse event; B, arm B; C, arm C; Cape, capecitabine; CNS, central nervous system; CPS, combined positive score; CT, chemotherapy; D, arm D; DDFS, distant disease-free survival; DFS, disease-free survival; Doce, docetaxel; DRFI, distant relapse-free interval; DRFS, distant relapse-free survival; EFS, event-free survival; ET, endocrine therapy; FEC, fluorouracil, epirubicin hydrochloride, cyclophosphamide; IDFS, invasive disease-free survival; Obs, observation; Ola, Olaparib; OS, overall survival; pCR, pathologic complete response; PD-L1, programmed death-ligand 1; Pembro, pembrolizumab; Pert, pertuzumab; PK, pharmacokinetics; Plac, placebo; PRO, patient-reported outcome; PTC, paclitaxel, trastuzumab, carboplatin; QoL, quality of life; RFI, recurrence-free interval; RFS, recurrence-free survival; Ribo, ribociclib; STEEP, standardized definitions for efficacy end points; T-DM1, trastuzumab emtansine; TDR, time to distant recurrence; Trast, trastuzumab.

a Including second primary non-breast cancer, per the STEEP definition.

### 3.1 NNT

**Fig. 1:**
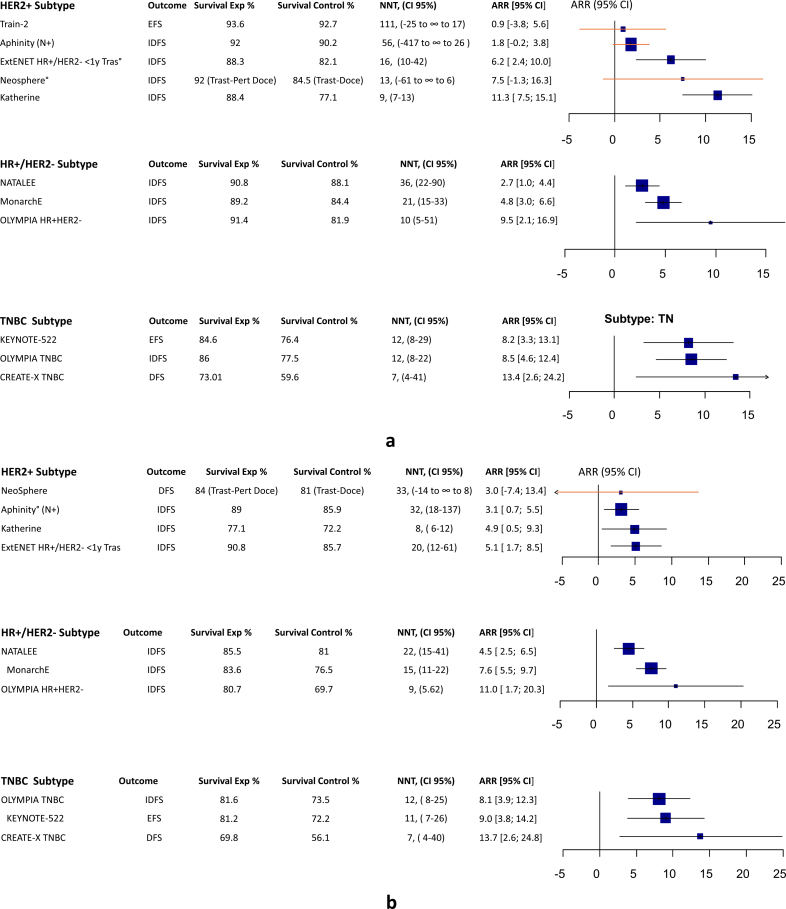
**Number needed to treat: IDFS/EFS/DFS.** **a: 3-year IDFS/EFS/DFS; b: 5-year IDFS/EFS/DFS.** °: Synthetic individual patient data (IPD) were generated from the digitized curves Abbreviations: ARR, absolute risk reduction; CI, confidence interval; Control, control arm; DFS, disease-free survival; EFS, event-free survival; Exp, experimental arm; HER2, Human epidermal growth factor receptor 2; HR, hormone receptor; IDFS invasive disease-free survival; ITT, intention-to-treat population; NNH, number needed to harm; NNT, number needed to treat; Tras, trastuzumab; y, years.

**Fig. 2:**
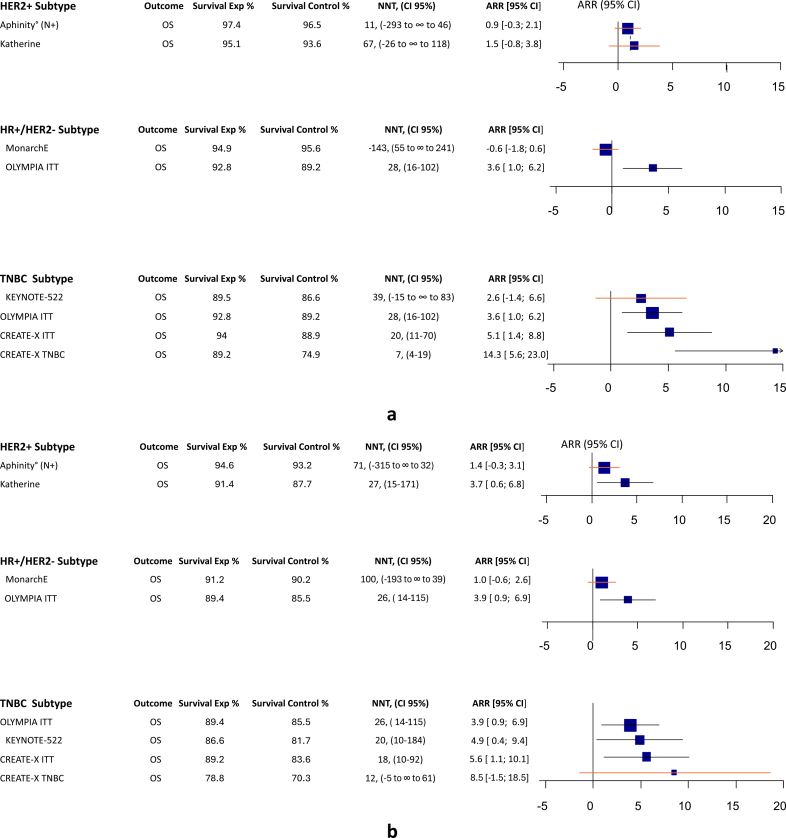
**Number needed to treat: OS.** **a: 3-year OS; b: 5-year OS.** °: Synthetic individual patient data (IPD)were generated from the digitized curves Abbreviations: ARR, absolute risk reduction; CI, confidence interval; Control, control arm; Exp, experimental arm; HER2, Human epidermal growth factor receptor 2; HR, hormone receptor; ITT, intention-to-treat population; NNH, number needed to harm; NNT, number needed to treat; OS, overall survival; TN, triple negative; y, years.

ARRs and NNTs with 95% CIs at 3 and 5 years are shown in Fig. 1, Fig. 2. Additional NNT estimates at other time points are provided in the Supplementary Materials (Supplementary Table 1). Moreover, OS NNT estimates from trials without statistically significant OS results are reported in the Supplementary Material, because their NNT confidence intervals necessarily crossed zero, and should not be interpreted as evidence of benefit (Supplementary Table 1).

#### 3.1.1 HR+/HER2− disease

The OlympiA [26] trial reported the most favorable NNT for IDFS at 3 years (10; 95% CI, 5-51), followed by monarchE [24] (21; 95% CI, 15–33), and NATALEE [25] (36; 95% CI, 22–90). At 5 years, the benefit remained consistent, with OlympiA [26] again showing the lowest NNT (9; 95% CI, 5–62), followed by monarchE [24] (15; 95% CI, 11–22) and NATALEE [25] (22; 95% CI, 15-41). For OS at 5 years, OlympiA [26] showed the clearest absolute benefit (26; 95% CI, 14–115) followed by monarchE [24] (100; 95% CI, −193 to ∞ to 39).

#### 3.1.2 HER2+ disease

KATHERINE [19] reported the most favorable NNT for IDFS at 3 years (9; 95% CI, 7–13), followed by NeoSphere [22] (13; 95% CI, −6 to ∞ to 66), ExteNET [23] (16; 95% CI, 10–42), APHINITY [20] (56; 95% CI, −417 to ∞ to 26) and TRAIN-2 [10] (111; 95% CI, −25 to ∞ to 17).

At 5 years, KATHERINE [19] (8; 95% CI, 6–12) and ExteNET [23] (20; 95% CI, 12–61) maintained consistent benefit, followed by APHINITY [20] (32; 95% CI, 18–137), and NeoSphere [22] (33; 95% CI, −14 to ∞ to 8). For OS at 5 years, KATHERINE [19] showed showed a statistically supported absolute benefit, with an NNT of 27 (95% CI, 15–171), followed by APHINITY [21] (71; 95% CI, −315 to ∞ to 32). OS data were not available for NeoSphere.

#### 3.1.3 Triple negative disease

At 3 years, the most favorable NNT for DFS was observed in the CREATE-X trial [11] (7; 95% CI, 4–41), followed by OlympiA [26] (12; 95% CI, 8–22) and KEYNOTE-522 [27] (12; 95% CI, 8–29) for IDFS and EFS, respectively. At 5 years, CREATE-X [11] demonstrated a sustained benefit (7; 95% CI, 4–40), with comparable results reported in KEYNOTE-522 [27] (11; 95% CI, 7–26) and OlympiA [26] (12; 95% CI, 8–25).

For OS, CREATE-X [11] reported the lowest NNT in the intention-to-treat (ITT) population (18; 95% CI, 10–92), followed by OlympiA [26] (26; 95% CI, 14–115) and KEYNOTE-522 [27] (20; 95% CI, 10–184).

### 3.2 NNH

NNHs were calculated for any-grade AEs, G ≥ 3 AEs, G5 AEs, AEs leading to TD, and the most frequently reported G ≥ 3 AE (Table 2).

**Table 2:** Reported adverse events in phase II-III trials included in the analysis.

Trial	Experimental arm	Control arm	Drug discontinuation in the Experimental arm (%)	Drug discontinuation in the Control arm (%)	Grade 5 AEs in the Experimental arm (%)	Grade 5 AEs in the Control arm (%)	Grade ≥3 AEs in the Experimental arm (%)	Grade ≥3 AEs in the Control arms (%)	Most common Grade ≥3 A E in the Experimental arm (%)	Most common Grade ≥3 AEs in the Control arm (%)	Most common any-grade AEs in the Experimental arm (%)	Most common any-grade AEs in the Control arm (%)
**KATHERINE** [19]	T-DM1	Trast	133 (18)	15 (2.1)	1 (0.1)^a^	0^a^	190 (25.7)	111 (15.4)	Decreased platelet count 27 (5.7)	Hypertension 9 (1.2)	Fatigue 366 (49.5)	Fatigue 243 (33.8)
**APHINITY** [20,32,33]	Pert + Trast + CT	Plac + Trast + CT	186 (7.3)	155 (6.2)	23 (1%)^c^	32 (1.3)^c^	1517 (64.2)	1379 (57.3)	Neutropenia 385 (16.3)	Neutropenia 377 (15.7)	/	/
**KEYNOTE-522** [27]	Pembro + CT	Plac + CT	216 (27.6)^a^	55 (14.1)^a^	4 (0.5)^a^	1 (0.3)^a^	604 (77.1)^a^	285 (73.3)^a^	Neutropenia 270 (34.6)^a^	Neutropenia 130 (33.2)^a^	Nausea 490 (62.7)^a^	Nausea 246 (63.2)^a^
**OlympiA** [26]	Ola	Plac	98 (10.8)	42 (4.6)	5 (<1%)	10 (1.1)	223 (24.5)	102 (11.3)	Anemia 79 (8.7)	Neutrophil count decreased 7 (0.8)	Nausea 520 (57.1)	Fatigue 248 (27.4)
**CREATE-X** [11]	Cape	Plac	101	0	0^a^	0^a^	/	/	Hand foot syndrome 49 (11.1)	Hypertransaminasemia 4 (0.8)	Hand foot syndrome 325 (73.4)	/
**ExteNET HR+ ≤1y** [23]	Neratinib	Plac	178 (27)^b^	30 (5)^b^	1 (0.1)^b^	0^b^	327 (49)^b^	76 (12)^b^	Diarrhea 261 (39)^b^	Diarrhea 7 (1)^b^	/	/
**monarchE** [31,34,35]	Abema + ET	ET	180 (6.4)	30 (1.1)	2^a^	0^a^	1289 (46.2) G3^b^	439 (15.7) G3^b^	Neutropenia 548 (19.6)^b^	Neutropenia 24 (0.9)^b^	/	/
**NATALEE** [25,36,37]	Ribo + ET	ET	509 (20)	124 (4.9)	0^a^	0^a^	1471 (58.2) G3 AEs	437 (17.9) G3 AEs	Neutropenia 1121 (44.4)	Liver related AE 31 (1.3)	Neutropenia 1587 (62.8)	Arthralgia 1083 (44.4)
**TRAIN-2** [10]	PTC + Pert	FEC + Trast + Pert	32 (15)	27 (12)	1	0	/	/	Neutropenia 118 (54)	Neutropenia 131 (60)	/	/
**NeoSphere** [22]	Trast + Doce (A)	Trast + Pert + Doce (B) Trast + Pert (C) Pert + Doce (D)	0	(B) 5 (5) (C) 8 (7) (D) 5 (5)	(B) 1 (1)^a^	(A) 0^a^	(B) 78 (73)	(A) 87 (81) (C) 75 (60) (D) 74 (79)	Neutropenia (B) 59 (55)	Neutropenia (A) 71 (66) (C) 40 (37) (D) 60 (64)	(B) Alopecia 73(68)	(A) Alopecia 80 (75) (C) Alopecia 59 (55) (D) Neutropenia 69 (73)

Abbreviations: A, arm A; Abema, abemaciclib; AE, adverse event; B, arm B; C, arm C; Cape, capecitabine; CT, chemotherapy; D, arm D; Doce, docetaxel; ET, endocrine therapy; FEC, fluorouracil, epirubicin hydrochloride, cyclophosphamide; G, grade; Obs, observation; Ola, Olaparib; Pembro, pembrolizumab; Pert, pertuzumab; Plac, placebo; PTC, paclitaxel, trastuzumab, carboplatin; Ribo, ribociclib; T-DM1, trastuzumab emtansine; Trast, trastuzumab; y, year.

a Treatment related AEs;/Not available.

b Treatment emergent AEs..

c Fatal AEs.

#### 3.2.1 HR+/HER2− disease

**Fig. 3:**
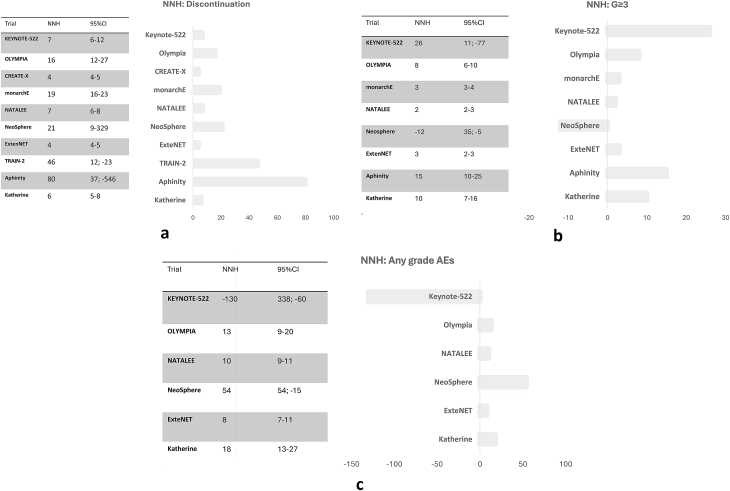
Number needed to harm. a: Discontinuation b: G ≥ 3 AEs c: Any grade AEs.

In patients with HR+/HER2− disease, the NATALEE [36] trial showed the highest toxicity risk, with NNHs of 10 (95% CI, 9-11) for any-grade AEs, 3 (95% CI, 2–3) for G ≥ 3 AEs, and 7 (95% CI, 6-8) for TD (Fig. 3).

#### 3.2.2 HER2+ disease

In patients with HER2+ disease, ExteNET [23] reported the lowest NNHs for any-grade AEs (8; 95% CI, 7–11), G ≥ 3 AEs (3; 95% CI, 2–3), and TD (4; 95% CI, 4–5), indicating the highest toxicity risk among the available HER2-directed strategies. KATHERINE [38] was also associated with substantial toxicity, with NNHs of 10 (95% CI, 7–16) for G ≥ 3 AEs and 6 (95% CI, 5-8) for TD (Fig. 3).

#### 3.2.3 Triple negative disease

In patients with Triple Negative disease, OlympiA [26] showed the lowest NNHs for any-grade AEs (13; 95% CI, 9–20) and G ≥ 3 AEs (8; 95% CI, 6–10). Additionally, CREATE-X [11] (4; 95% CI, 4–5) and KEYNOTE-522 [27] (7; 95% CI, 6–12) were associated with the highest risk of TD (Fig. 3). Detailed NNHs for most frequent G ≥ 3 AEs and for G5 AEs are provided in Supplementary Table 2.

## 4 Discussion

In this study, we analysed RCTs evaluating therapeutic escalation strategies in patients with intermediate-to high-risk eBC and derived NNT and NNH to characterize the balance between treatment benefit and harm. Overall, our findings highlight substantial heterogeneity in benefit–risk profiles across disease subtypes and treatment strategies, with considerable variability in toxicity burden. As intuitive absolute measures, NNT and NNH may facilitate clinical interpretation and support patient-centered discussions by enabling the presentation of therapeutic trade-offs in a numerically grounded but user-friendly format. These metrics may also provide a simple framework for health-economic considerations, by linking the number of patients treated to the number of clinical events prevented or adverse events incurred [39,40]. For example, stating that “one in 12 patients treated will benefit over five years” may be more impactful and comprehensible than citing a “7–8% absolute risk reduction”. However, it is important to acknowledge that the NNT and NNH estimates are time-dependent and may not fully capture the dynamics of treatment effects. Hazard ratios derived from time-to-event analyses reflect the relative risk over the entire follow-up period, whereas NNTs provide a snapshot of the absolute benefit at a fixed time point, potentially overlooking more nuanced temporal patterns. This discrepancy may lead to divergent interpretations, particularly when comparing heterogeneous trials with varying follow-up durations. For example, treatments with delayed efficacy (e.g., immunotherapy) or late toxicities (e.g., CDK4/6 inhibitors) may have their benefit or harm underestimated (or overestimated) when assessed only at early time points. Therefore, meaningful interpretation of NNT and NNH requires standardized reporting of the outcome, comparator and time horizon, particularly when these metrics are used for cross-trial comparisons [41].

To partially address this issue, we reported the NNT values separately by breast cancer subtype at fixed time points (3 and 5 years), thereby illustrating how the magnitude of benefit may evolve over time. Among the included trials, OlympiA [26], CREATE-X [11], and KEYNOTE-522 [27] in patients with TNBC disease, and KATHERINE [19] in patients with HER2+ disease, showed consistent NNT values over time, suggesting both early and sustained clinical benefits. However, the distinct biological behaviors of BC subtypes substantially affect the interpretation of these measures. TNBC is typically associated with a high risk of early recurrence, concentrating treatment effects within the first years, whereas HR+/HER2– tumors tend relapse later, with a persistent long-term risk of recurrence [42]. These biological patterns are critical for the proper contextualization of NNT and NNH estimates. Consequently, early NNTs estimates may adequately reflect treatment benefit in TNBC, while in HR+/HER2− disease they may underestimate the magnitude of long-term benefit, underscoring the importance of extended follow-up.

In particular, in patients with HR+/HER2– eBC, both monarchE [24] and NATALEE [25] trials demonstrated a progressive reduction in NNT for IDFS even after treatment discontinuation, suggesting a potential carry-over effect, defined as a persistent or increasing treatment benefit after discontinuation of active therapy and previously described for adjuvant endocrine therapies [43,44]. At the same time, these trials highlighted the relevant toxicity burden associated with CDK4/6 inhibitor-based strategies. Neutropenia was the most frequent G ≥ 3 AE, and TD rates were considerable. In high-risk early TNBC, treatment options commonly include capecitabine, pembrolizumab, or olaparib [8,28]. The CREATE-X [11] trial demonstrated the most favorable 5-year NNT for OS; however, only ∼31% of patients had TNBC, none had received prior carboplatin or neoadjuvant immunotherapy, and capecitabine was associated with substantial toxicity, with hand-foot syndrome being the most frequent severe AE. Moreover, the comparator consisted of observation without any additional systemic treatment, which may partially account for to the relatively low NNT and limits direct comparability with more contemporary escalation strategies. By contrast, findings from OlympiA [26] and KEYNOTE-522 [27], which more closely reflect current patient profiles and modern treatment algorithms, may be more representative of present clinical practice. In patients with HER2+ disease, the KATHERINE [19] trial demonstrated consistent NNTs for both IDFS and OS, although the NNH for TD reflected a clinically relevant incidence of toxicity. However, the study population did not include patients who had received neoadjuvant pertuzumab. In contrast, the NeoSphere [22] and APHINITY [20] trials evaluated the addition of pertuzumab in the neoadjuvant and adjuvant settings, respectively, achieving comparable benefits in terms of NNT for IDFS, with a reassuring safety profile, particularly regarding grade ≥3 AEs and TD. On the contrary, in the ExteNET [23] trial, despite a modest reduction in recurrence risk, reflected by NNT estimates, escalation therapy with neratinib was associated with substantial gastrointestinal toxicity, resulting in unfavorable NNHs for both G ≥ 3 AEs and TD. These findings raise concerns regarding the clinical applicability of this strategy in contemporary practice.

Despite the revision of the Consolidated Standards of Reporting Trials (CONSORT) statement, which recommends reporting RCT results using NNT and NNH for both binary and time-to-event outcomes [45], these metrics remain underused in the medical literature [46]. Moreover, when reported, CIs for NNTs are often omitted, which represents an important limitation [18].

We acknowledge the inherent limitations of the NNT and NNH estimates. In several cases, particularly NNTs for OS and NNHs for all-grade AEs, CI were wide and/or crossed zero, reflecting uncertainty arising from small absolute differences between treatment arms. In particular, a major methodological caveat concerns the interpretation of estimates derived from comparisons in which the 95% CI of the underlying absolute risk difference, either ARR or ARI, includes zero [47]. In such cases, the data are compatible with both benefit and harm, and the corresponding NNT or NNH point estimate should not be interpreted in isolation as quantitative evidence of treatment effect or toxicity. We therefore reported these estimates using the Altman convention [18], which makes the bidirectional uncertainty explicit, and caution readers against drawing conclusions about the magnitude of benefit or harm from non-significant comparisons, particularly for OS endpoints, where several included trials had not yet reached statistical significance at the time of the most recent reported analysis.

Accordingly, although easily calculated and intuitively appealing, NNT and NNH should be considered alongside relative risk measures and relevant clinical context. In particular, these metrics are influenced by both the choice of comparator and the baseline risk of the study population. Trials employing less intensive control arms may yield more favorable NNT estimates, as in CREATE-X trial [11], where the comparator for TNBC consisted of observation alone. Furthermore, ongoing improvements in standard therapies progressively reduce baseline recurrence risk, potentially leading to higher NNTs over time even for effective interventions, particularly for OS outcomes. Because NNT is inherently dependent on baseline risk, patients at higher risk of recurrence tend to derive greater absolute benefit, as observed for the BRCA-mutated population enrolled in the OlympiA trial [26]. NNH estimates similarly have limitations, as they may not fully capture qualitative aspects of toxicity, including chronic treatment burden and quality-of-life impact, which are particularly relevant in the curative setting. Moreover, beyond their statistical interpretation, NNT and NNH should also be considered within the broader context of benefit–harm communication in oncology. The way treatment benefits and toxicities are presented to patients, including the format, framing, timing, and amount of information provided, may influence understanding, emotional response, and ultimately treatment choices [48,49]. In particular, numerical or visual formats may improve comprehension compared with purely verbal descriptions, while excessive or poorly timed information on treatment consequences may increase distress and undermine confidence [50]. In this context, NNT and NNH may help translate absolute benefit and harm into more clinically intuitive estimates, but they should not be presented as stand-alone measures. On the contrary, their use in clinical communication should remain individualized, avoiding both oversimplification and information overload. Additionally, they should support individualized discussions in which the numerical estimate is interpreted in light of the clinical relevance of the endpoint, the nature and burden of toxicity, the uncertainty of the estimate, and the patient's values and preferences. This study has several limitations that should be acknowledged. Heterogeneity across trials in terms of study design, patient populations, median follow-up, treatment duration, and endpoint definitions limits direct cross-trial comparability. In particular, DFS, IDFS, and EFS are distinct intermediate endpoints and should not be considered fully interchangeable. Accordingly, efficacy endpoints were extracted and reported as defined in each individual trial, as summarized in Supplementary Table 3. Additionally, incomplete and variable AE reporting across trials, including differences in the reporting of AEs, treatment-related AEs, and treatment-emergent AEs, may have affected the accuracy of NNH estimates. Overall, these limitations reflect the heterogeneity of the available evidence and could not be fully harmonized across studies.

## 5 Conclusions

Our analysis highlights the value of NNT and NNH, as intuitive metric to characterize the benefit–risk balance of therapeutic escalation strategies in patients with intermediate-to high-risk eBC. By translating complex statistical results into clinically interpretable estimates, these measures may enhance both scientific communication and evidence-based decision-making. However, their interpretation requires careful consideration of trial design, patient population, follow-up duration, and the temporal dynamics of treatment efficacy and toxicity.

CI must always accompany these estimates to convey statistical precision and to mitigate the risk of misleading conclusions. Importantly, while NNT and NNH, provide a useful quantitative framework, they should complement, not replace, clinical judgment, integration of biological risk factors, and patient preferences.

In this regard, future studies incorporating patient partners or patient advocacy groups could help clarify how patients interpret and value these summary measures when weighing treatment benefit against potential harm. When applied responsibly, these measures can enhance shared decision-making and contextualize the trade-offs between therapeutic benefit and harm. Conversely, their presentation without appropriate contextualization risks oversimplifying the evidence and misrepresenting the true clinical implications of treatment.

## Ethical approval

This study did not require ethical approval in accordance with the journal's policy and applicable regulations.

## Funding source

This work was supported by the Italian Ministry of Health (Ricerca Corrente).

## CRediT authorship contribution statement

**Roberto Buonaiuto:** Conceptualization, Data curation, Formal analysis, Methodology, Resources, Writing – original draft, Writing – review & editing. **Federica Pia Mangiacotti:** Data curation, Writing – original draft, Writing – review & editing. **Fabiola Giudici:** Data curation, Formal analysis, Methodology, Writing – original draft. **Margherita Tafuro:** Data curation, Writing – original draft, Writing – review & editing. **Roberta Scarfetta:** Conceptualization, Data curation, Writing – original draft, Writing – review & editing. **Alessandra Longobardi:** Writing – review & editing. **Martina Pagliuca:** Writing – review & editing. **Aldo Caltavituro:** Writing – review & editing. **Luca Arecco:** Writing – review & editing. **Evandro de Azambuja:** Writing – review & editing. **Mario Giuliano:** Writing – review & editing. **Grazia Arpino:** Writing – review & editing. **Lucia Del Mastro:** Writing – review & editing. **Carmine De Angelis:** Methodology, Supervision, Writing – review & editing. **Michelino De Laurentiis:** Methodology, Supervision, Writing – review & editing. **Fabio Puglisi:** Data curation, Formal analysis, Methodology, Supervision, Validation, Visualization, Writing – review & editing.

## Declaration of competing interest

The authors declare the following financial interests/personal relationships which may be considered as potential competing interests: **FP** reports research grants from AstraZeneca, Eisai, and Roche. FP has received consulting fees from AstraZeneca, Daiichi Sankyo, Eli Lilly, Menarini, MSD, Novartis, Pfizer, and Roche; and payments/honoraria from AstraZeneca, Daiichi Sankyo, Eli Lilly, Exact Sciences, Gilead, Italfarmaco, Menarini, MSD, Novartis, Pfizer, and Roche. FP has served in advisory roles for Amgen, Daiichi Sankyo, Eli Lilly, Gilead, GSK, Novartis, Pfizer, and Roche. FP has received travel support and accommodation from AstraZeneca, Daiichi Sankyo, Menarini, Pfizer, and Roche, and has participated in advisory boards for AstraZeneca, Daiichi Sankyo, Eli Lilly, Exact Sciences, Menarini, MSD, Novartis, Pfizer, and Roche. **MP** reports institutional funding from Gilead and travel reimbursement from Ipsen. **LDM** reports grants from Eli Lilly, Novartis, Roche, Daiichi Sankyo, Seagen, AstraZeneca, Gilead, Pierre Fabre, Pfizer, Menarini Stemline, and MSD. LDM has received consulting fees from Novartis, Roche, Daiichi Sankyo, Eli Lilly, Gilead, Pfizer, Menarini Stemline, Olema, and AstraZeneca; and honoraria from Eli Lilly, Novartis, Roche, Daiichi Sankyo, Seagen, AstraZeneca, Gilead, Pierre Fabre, Pfizer, Menarini Stemline, MSD, Exact Sciences, GSK, Ipsen, and Eisai. LDM has received travel grants from Roche, Gilead, Menarini Stemline, AstraZeneca, and Daiichi Sankyo. LDM has served on advisory boards for Eli Lilly, Novartis, Roche, Daiichi Sankyo, Seagen, AstraZeneca, Gilead, Pierre Fabre, Pfizer, Menarini Stemline, MSD, Exact Sciences, GSK, Ipsen, and Eisai. LDM reports receipt of equipment from FoundationOne.**MDL** reports relationships with Roche, Novartis, Takeda, Eli Lilly, Pierre Fabre, AstraZeneca, MSD, Seagen, Gilead, Daiichi Sankyo, Tomalab, Genetic, Pfizer, Menarini, Sophos, Istituto Gentili, Sanofi, Ipsen, GSK, and Exact Sciences, including consulting/advisory roles and travel reimbursement. **MG** reports relationships with AstraZeneca, Daiichi Sankyo, Eisai, Gilead, Celgene, Exact Sciences, Eli Lilly, MSD, Novartis, Pfizer, Roche, and Seagen, including consulting/advisory roles and travel reimbursement. **GA** reports relationships with Roche, Pfizer, Eli Lilly, MSD, AstraZeneca, and Novartis, including consulting/advisory roles and research funding grants. PV has received honoraria and served in advisory roles for AstraZeneca, Daiichi Sankyo, Eli Lilly, Gilead, Incyte, Istituto Gentili, Novartis, Pfizer, Roche, Seagen, and Teva. PV has received travel support and accommodation from Daiichi Sankyo, Eli Lilly, and Novartis. PV reports research funding to the institution from Novartis and AstraZeneca.**CDA** reports consulting or advisory relationships with Novartis, GSK, Eli Lilly, and Pfizer. **EDA** reports honoraria and/or advisory board participation from Roche/GNE, Novartis, Seagen, Zodiac, Libbs, Pierre Fabre, Lilly, AstraZeneca, MSD, and Gilead Sciences; travel grants from AstraZeneca and Gilead; institutional research grants from Roche/GNE, AstraZeneca, GSK/Novartis, Gilead Sciences, and Seagen/Pfizer; and non-financial roles as ESMO Director of Membership (2023–2025) and BSMO President (2023–2026). **LA** reports travel grants from AstraZeneca and institutional research funding from Gilead Sciences. All other authors declare no competing financial interests or personal relationships that could have influenced the work reported in this paper.
