# Supplementary material for: Strengths and pitfalls of NNT and NNH in early breast cancer escalation trials
Source: Breast. 2026 May 29;88:104820. doi: 10.1016/j.breast.2026.104820 (PMC13277489; doi:10.1016/j.breast.2026.104820)
Supplement: Multimedia component 1 [file mmc1.docx]

| **Endpoint** | **Trial** | **Year** | **% Experimental arm** | **% Control arm** | **n Experimental arm** | **n Control arm** | **NNT** | **95% CI** |
| --- | --- | --- | --- | --- | --- | --- | --- | --- |
| IDFS | monarchE | 2 | 92.7 | 89.9 | 2808 | 2829 | 35 | 23-75 |
| IDFS | monarchE | 4 | 86 | 80 | 2808 | 2829 | 17 | 12-24 |
| IDFS | MonarchE | 6 | 80 | 74.8 | 2808 | 2829 | 17 | 12-27 |
| IDFS | MonarchE | 7 | 77.4 | 70.9 | 2808 | 2829 | 15 | 11-24 |
| IDFS | NATALEE | 4 | 88.5 | 83.6 | 2549 | 2552 | 20 | 15-33 |
| IDFS | OlympiA (HR+/HER2-) | 2 | 91.4 | 81.9 | 168 | 157 | 10 | 6-47 |
| IDFS | OlympiA (HR+/HER2-) | 4 | 82.9 | 72 | 168 | 157 | 9 | 5-55 |
| IDFS | OlympiA (HR+/HER2-) | 6 | 77.5 | 67.7 | 168 | 157 | 10 | 5-916 |
| IDFS | OlympiA (TN) | 2 | 89.3 | 81.3 | 751 | 757 | 13 | 9-22 |
| IDFS | OlympiA (TN) | 4 | 83.1 | 75.3 | 751 | 757 | 13 | 8-27 |
| IDFS | OlympiA (TN) | 6 | 80 | 70.8 | 751 | 757 | 10 | 7-19 |
| IDFS | KATHERINE | 7 | 80.8 | 67.1 | 743 | 743 | 7 | 5-11 |
| IDFS | APHINITY (N+) | 6 | 88 | 83.6 | 1503 | 1502 | 23 | 14-52 |
| IDFS | APHINITY (N+) | 8 | 86.1 | 81.2 | 1503 | 1502 | 20 | 13-44 |
| IDFS | ExteNET (HR+/HER2+  <1year) | 2 | 95.3 | 90.8 | 670 | 664 | 16 | 12-35 |

| IDFS | ExteNET (HR+/HER2+  >1year Trast) | 2 | 97.4 | 94.4 | 146 | 151 | 33 | -25 to∞ to 17 |
| --- | --- | --- | --- | --- | --- | --- | --- | --- |
| IDFS | ExteNET (HR+/HER2+  >1year Trast) | 5 | 93 | 91.7 | 146 | 151 | 77 | -15 to ∞ to 77 |
| OS | MonarchE | 6 | 89.2 | 87.9 | 2808 | 2829 | 77 | -274 to ∞ 33 |
| OS | MonarchE | 7 | 86.8 | 85 | 2808 | 2829 | 56 | -5580 to ∞ to 28 |
| OS | OlympiA ITT | 2 | 95.0 | 92.8 | 921 | 915 | 45 | 22-NA |
| OS | OlympiA ITT | 4 | 90.4 | 87.2 | 921 | 915 | 31 | 16-320 |
| OS | OlympiA ITT | 6 | 87.5 | 83.2 | 921 | 915 | 23 | 13-94 |
| OS | KEYNOTE-522 | 4 | 87.8 | 83.5 | 784 | 390 | 23 | 11- 765 |
| OS | KATHERINE | 7 | 89.1 | 84.4 | 743 | 743 | 7 | 5-11 |
| OS | APHINITY (N+) | 10 | 89.6 | 86.9 | 1503 | 1502 | 37 | 20-252 |
| OS | ExteNET (HR+/HER2+  <1year Trast) | 2 | 99.1 | 98.5 | 670 | 664 | 166 | -154 to ∞ to 52 |
| OS | ExteNET (HR+/HER2+  <1year Trast) | 8 | 91.5 | 89.4 | 670 | 664 | 48 | -73 to ∞ to 20 |
| OS | ExteNET (HR+/HER2+  <1year Trast)* | 3 | 97.7 | 97 | 670 | 664 | 143 | -41 to ∞ to 93 |
| OS | ExteNET (HR+/HER2+  <1year Trast)* | 5 | 95 | 92.5 | 670 | 664 | 40 | -896 to ∞ to 19 |
| OS | TRAIN-2 | 3 | 98.2 | 97.7 | 219 | 219 | 200 | -39to ∞ to 27 |
| OS | NATALEE* | 3 | 97 | 96.2 | 2549 | 2552 | 125 | -503 to ∞ to 55 |
| OS | NATALEE* | 5 | 94.1 | 92.5 | 2549 | 2552 | 62 | 34-441 |

# Supplementary Table 1. Numbers Needed to Treat with 95% Confidence Intervals at additional time points.

Abbreviations: CI confidence interval; HR, hormone receptor; HER2 Human epidermal growth factor receptor 2; IDFS, invasive disease-free survival; ITT, intention-to-treat population; N, lymph node; n, number; NNT, number needed to treat; OS, overall survival; Trast, trastuzumab. *: Synthetic individual patient data (IPD) were generated from the digitized curves

| **HER2+ subgroup** | | | | | | |
| --- | --- | --- | --- | --- | --- | --- |
| **Trial** | **Experimental arm** | **Control arm** | **G5 AEs** | **95% CI** | **Most common G≥3 AEs** | **95% CI** |
| KATHERINE | TDM-1 | Plac | 740 | 131; -245 | 69 | 34-256 |
| APHINITY | Pert | Plac | -268 | 416; -100 | -2.8 | -2.5; -3.1 |
| TRAIN-2 | No Antra | Antra | 222 | 40; -76 | -14 | 44; -6 |
| ExteNET | Neratinib | Plac | 662 | 117; -225 | 2.6 | 2.4-2.9 |
| NeoSphere | Trast+Pert+Doce | Trast+Doce | 111 | -38; 20 | -9 | -4; 57 |
| **TN subgroup** | | | | | | |
| CREATE-X ITT | Cape | Plac | - | - | 9 | 7-12 |
| KEYNOTE-522 | Pembro | Plac | 394 | 93; -103 | 6 | 5-8 |
| OlympiA ITT | Ola | Plac | -179 | 303; -65 | 13 | 10-16 |
| **HR+/HER2- subgroup** | | | | | | |
| monarchE | Abema+ET | ET | 1395* | 383; -1335 | 5.3 | 4.9-5.8 |
| NATALEE | Ribo+ET | ET | * | * | 2.3 | 2.2-2.5 |
| CREATE-X ITT | Cape | Plac | - | - | 9 | 7-12 |
| OlympiA ITT | Ola | Plac | -179 | 303; -65 | 13 | 10-16 |

# Supplementary Table 2. Summary of grade 5 adverse events and most common grade ≥3 adverse events with 95% Confidence Intervals.

Abbreviations: Abema, abemaciclib; AE, adverse event; Antra, anthracyclines; Cape, capecitabine; CI, confidence interval; Doce, docetaxel; ET, endocrine therapy; HR, hormone receptor; HER2 Human epidermal growth factor receptor 2; G, grade; ITT, intention-to-treat population; Ola, Olaparib; Pembro, pembrolizumab; Pert, pertuzumab; Plac, placebo; Ribo, ribociclib; T-DM1, trastuzumab emtansine; TN, triple negative; Trast, trastuzumab.

*Treatment related;

| **Trial** | **Endpoint** | **Definition** |
| --- | --- | --- |
| KHATERINE | IDFS | IDFS was defined as the time from randomization until the date of the first occurrence of one of the following events (hereafter referred to as invasive-disease events): recurrence of ipsilateral invasive breast tumor, recurrence of ipsilateral locoregional invasive breast cancer, contralateral invasive breast cancer, a distant disease recurrence, or death from any cause. |
| APHINITY | IDFS | IDFS was defined as the time from randomization until the date of the first occurrence of one of the following events (hereafter referred to as invasive-disease events): recurrence of ipsilateral invasive breast tumor, recurrence of ipsilateral locoregional invasive disease, a distant disease recurrence, contralateral invasive breast cancer, or death from any cause. |
| KEYNOTE-522 | EFS | EFS, was defined as the time from randomization to disease progression that precludes definitive surgery, local or distant recurrence, a second primary cancer, or death from any cause, whichever occurred first |
| OlympiA | IDFS | IDFS was defined as the time from randomization until the date of first occurrence of one of the following events: ipsilateral invasive breast tumor, locoregional invasive disease, distant recurrence, contralateral invasive breast cancer, second primary invasive cancer, or death from any cause |
| CREATE-X | DFS | DFS was defined as the time from randomization to recurrence, the development of a second cancer, or death from any cause. Secondary end points included overall survival, which was defined as the time from randomization to death from any cause |
| ExteNET | IDFS | IDFS was defined as the time from randomization to first occurrence of invasive ipsilateral tumor recurrence, invasive contralateral breast cancer, local/regional invasive recurrence, distant recurrence, or death from any cause. |

| monarchE | IDFS | IDFS was defined as the time from the randomization to the date of first occurrence of ipsilateral invasive breast tumor recurrence, local/regional invasive breast cancer recurrence, distant recurrence, death attributable to any cause, contralateral invasive breast cancer, or second primary non breast invasive cancer. |
| --- | --- | --- |
| NATALEE | IDFS | IDFS was defined as the time from randomization until the date of the first occurrence of one of the following events (hereafter referred to as invasive-disease events): Invasive IBTR, Local-regional invasive recurrence, Distant recurrence, Death from non-breast cancer cause, Death from breast cancer, Death from unknown Cause, Invasive contralateral breast cancer, Second primary invasive cancer (non-breast) |
| TRAIN-2 | EFS | EFS is defined as the time from randomization to disease progression resulting in inoperability, recurrence (contralateral ductal carcinoma in situ excluded), secondary primary malignant neoplasms, or death by any cause |
| NeoSphere | DFS | DFS was defined as time from the first date of no disease (i.e., date of surgery) to the first documentation of progressive disease or death |

# Supplementary Table 3. Definitions of endpoints in phase II–III trials included in the analysis.

Abbreviations: DFS, disease-free survival; EFS, event-free survival; IDFS, invasive disease-free survival; RECIST, Response Evaluation Criteria In Solid Tumors.
